# Follow-up Study Tracking Children’s Development from Preschool till Middle School

**DOI:** 10.1192/j.eurpsy.2023.655

**Published:** 2023-07-19

**Authors:** K. Nomura, K. Yokoyama

**Affiliations:** Nagoya university, Nagoya, Japan

## Abstract

**Introduction:**

Screening for early detection of health issues and support are provided to children needing developmental support. In Japan, a significant percentage of infants requiring support are identified during health checkups. Sometimes, however, problems are first observed when children are of school age. It is, therefore, important to identify the age at which children need early support.

**Objectives:**

Of the children born in 2005 in Kanie-cho, in Japan, 106 participated in the survey at all time points: age 5, first grade, fifth grade, and eighth grade.

**Methods:**

The medical checkup results of the participants at age 5 were used to determine who among them needed support After entering school, the participants who scored less than 70 points on the Children’s Global Assessment Scale, where their adjustment was assessed based on the interview with the homeroom teacher, were considered maladjusted.

**Results:**

The results are presented in Table 1.

Thirty participants needed supports at age 5; of these, 20 (66.7%) were maladjusted at any point in their school years—19 (95%) in the first grade, 14 (70%) till the fifth grade, and five (25%) till the eighth grade.

Of the 76 participants who did not need support in early childhood, 24 (31.6%) were maladjusted at some point in their school years—nine (37.5%) experienced maladjustment in the first grade, but none of them continued to be maladjusted till the fifth grade, and 14 (58.3%) who were not maladjusted in the first grade experienced it in the fifth or eighth grade (adolescents).

Thus, the participants maladjusted in their school years were categorized as follows:

1. The developmental disorders group (experiencing maladjustment throughout since early childhood): 19

2. The “first grade problem” group (experiencing transient problems only in the first grade): 9

3. The adolescent group (experiencing problems during adolescence): 14

**Image:**

**Figure:**
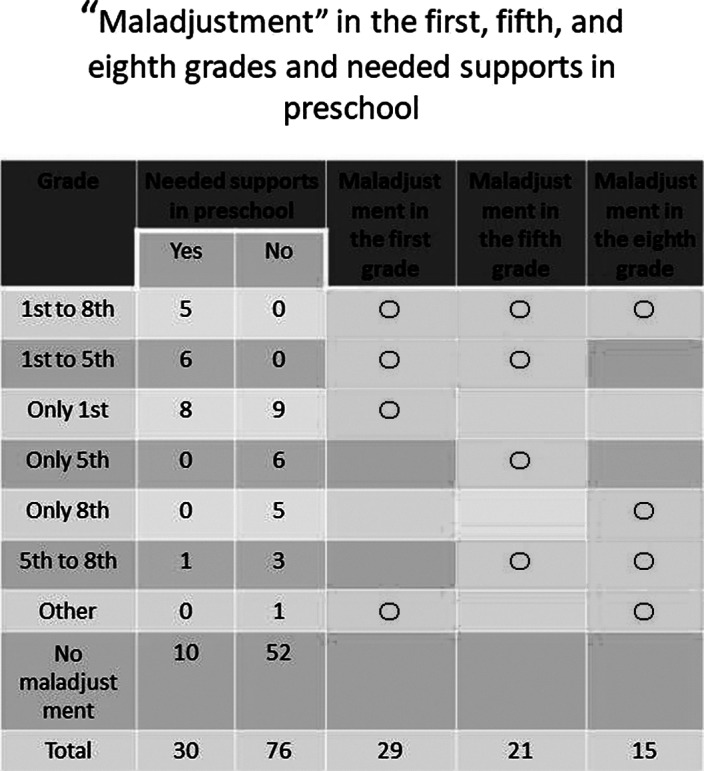

**Conclusions:**

Since maladjusted children with developmental disabilities are identified in early childhood, support can be provided before they reach school age. Many children with developmental disabilities improve their adjustment as they grow up. It is thus advisable to take a long-term perspective in dealing with problematic behaviors.

From late school age to adolescence, problems unrelated to developmental disabilities emerge. By listening to the child’s upbringing, it may be possible to ascertain whether or not the problem stems from a developmental disability.

**Disclosure of Interest:**

None Declared

